# Effect of operational parameters on spray performance and drift characteristics of a UAV-based agrochemical application in pigeon pea crop to control thrips

**DOI:** 10.3389/fpls.2025.1570272

**Published:** 2025-05-30

**Authors:** Gatkal N. R., Nalawade S. M., Shelke M. S., Deshmukh M. S., Bhanage G. B., More N. M., Ramesh K. Sahni, Marcel Mikeska

**Affiliations:** ^1^ Department of Farm Machinery and Power Engineering, Dr. Annasaheb Shinde College of Agricultural Engineering and Technology, Mahatma Phule Krishi Vidyapeeth, Ahmednagar, India; ^2^ Department of Soil Science and Agricultural Chemistry, College of Agriculture, Vasantrao Naik Marathwada Krushi Vidyapeeth, Parbhani, India; ^3^ Centre for Advanced Agricultural Science and Technology for Climate Smart Agriculture and Water Management (CAAST-CSAWM), Dr. Annasaheb Shinde College of Agricultural Engineering and Technology, Mahatma Phule Krishi Vidyapeeth, Ahmednagar, India; ^4^ ICAR—Central Institute of Agricultural Engineering, Bhopal, India; ^5^ VSB-Technical University of Ostrava, CEET, ENET Centre, Ostrava-Poruba, Czechia

**Keywords:** UAV spraying, pigeon pea, drift, flight speed, flight height, droplet density, droplet size, coverage

## Abstract

In the past few years, UAV application in agriculture has increased significantly due to higher efficiency and safety, simple operation, reduced labor requirements, and saving chemicals as compared to conventional sprayers. The UAVs are widely used in agriculture, providing flexibility and more profit to farmers. In recent years, research has been conducted on various operational parameters of UAV, and there has been no experiment or study on the effect of operational parameters and drift characteristics of UAV and control of thrips in pigeon pea crop. Therefore, this study evaluated the effect of the operational parameters of a UAV-based spraying system on the performance of agrochemical application in pigeon pea crop to control thrips. A field study was conducted to determine the performance parameters in terms of droplet deposition density, droplet size, coverage, spray deposition, and relative span at different flight heights (1.5, 2, and 2.5 m above the crop canopy) and flight speeds (2, 2.5, and 3 m/s). Water-sensitive papers (WSPs) were placed at three canopy zones (bottom, middle, and top zones) of the pigeon pea plant. The maximum droplet density, droplet size, coverage, spray deposition, and relative span factor at the top, middle, and bottom canopy zones were 54.00, 50.17, and 46.33 droplets/cm²; 244.80, 239.88, and 235.37 µm; 10.53%, 10.09%, and 9.78%; 0.764, 0.714, and 0.672 µl/cm²; and 0.98, 0.96, and 0.93, respectively, at a flight height of 1.5 m and a flying speed of 2 m/s. Spray deposition was negligible in off-target zones. The field capacity, field efficiency, and application rate of the UAV were found to be 2.62 ha/h, 60.64%, and 77.86 L/ha, respectively. The maximum control efficacy of thrips on the top, middle, and bottom pigeon pea canopy was 92.45%, 90.12%, and 88.11% after 10 days of spraying experiment. This study provides recommendations for optimal operating parameters (height: 1.5 m and speed: 2 m/s) for efficient agrochemical application, benefiting manufacturers, farmers, and UAV operators for more effective and efficient spraying on pigeon pea crops.

## 1 Introduction

In agriculture, pesticides are used to control insects, diseases, and weeds that threaten crop and decline yield. To achieve maximum crop production, modern agriculture includes pesticides, which are hazardous for the environment, soil, water bodies, and human health (Hafeez et al., 2022; Kumar et al., 2021). Among the most common methods used in agriculture to apply pesticides are fumigation, foliar spraying, and soil application. The most popular and widely accepted strategy for applying pesticides is by using different plant protection devices that produce tiny droplets (Zhang et al., 2014; Jyoti et al., 2022; Sahni et al., 2022).

Pigeon pea also called red gram (*Cajanus cajan* L. Millsp.) is the second most important pulse crop after gram. Traditional pesticide spray application techniques apply chemicals inappropriately and result in spray uniformity, deposition, coverage, and spray deposition. These factors raise the price of insecticide and lead to environmental contamination and increased labor costs (Wang et al., 2020). Conventional sprayers lead to inappropriate application of insecticides when crop height is higher. Knapsack sprayers and boom sprayers are commonly used for insecticide application in pigeon pea crop. During knapsack spraying, crop canopy is damaged due to the operator walking between the crop rows while tractor-operated boom spraying damages the crop plant, rolls the plant, and sometimes drags the fruit branches during insecticide application (Dou et al., 2022). Insecticide application on pigeon pea crop through UAV is one of the solutions to managing crops effectively and efficiently because UAV sprays above the crop without damaging crop plants and saves water, and insecticide utilization rate is higher (Dou et al., 2022).

UAVs are a newly advanced technology and gained popularity in various applications, such as pesticide sprays, because of their potential benefits, like spraying applications for higher height crops, minimum labor requirement by replacing backpack sprayers, and the use of inaccessible fields (2018). These UAVs can apply insecticides, pesticides, herbicides, and fertilizers more effectively and efficiently. By spraying specific areas, they improve crop growth, mitigate waste, and make farming more resilient to climate change (Li et al., 2024). These UAVs provide accurate, precise pesticide doses, minimize waste, reduce environmental impacts, and improve crop productivity (Positano et al., 2024). The effectiveness of these UAVs is dependent on the optimization of their operational parameters. The various parameters, including flying height, flight speed, type of nozzle, droplet size, and rate of application, must all be accurately provided with uniform coverage and effective pest control. Optimizing operational parameters is essential for maximizing pesticide effectiveness and enhancing the nutritional value of crops. UAVs are equipped with sophisticated features in autonomous spraying devices, such as autonomous path planning, a terrain-following radar module (auto altitude adjustment based on crop height), a break point to continue spraying (Wang et al., 2021), a high-precision obstacle avoidance radar, a spray chemical empty indicator, a spray task list, a battery level warning, and high-accuracy real-time kinematics (RTK) location, which improves operational reliability, effectiveness, precision, accuracy, and ease of use (Yang et al., 2018). Moreover, precise spraying can minimize crop stress from pests, diseases, and climatic conditions, hence enhancing resilience. The effectiveness of pesticide application can have a significant impact on stress resilience, which is another essential component of crop health. The increase in vulnerability to stresses is usually encountered in plants that experienced late maturing crops and lower nutritional characteristics. The operating parameters of UAVs, including flight height, flying speed, payload, and design, strongly influence droplet penetration and distribution (Yan et al., 2023). The use of UAVs (multi-rotor) for chemical spraying generates strong downwash, which helps to reduce crop disturbance and increase chemical penetration (Lan et al., 2021). The generated downwash airflow of the UAV rotors can result in significant plant velocity distribution while spraying. This improves spray droplet atomization and deposition on the crop surface. Spray droplet velocity improves spray swath, deposition, and drift, altering the operation’s performance (Li et al., 2018).

Several researchers and scientists across the globe studied the various operational parameters of UAVs, such as flight height, flying speed, nozzle type, flow rate, nozzle pressure, payload, and UAV model, to enhance the deposition rate and minimize the drift of chemical solution on the target zone. The UAV efficacy is strongly influenced by spray volume, size of droplet, rate of deposition, and weather parameters like temperature, wind speed, wind direction, relative humidity, and rain (Paul et al., 2024). Qin et al. (2016) studied the influence of flying height (0.8 to 1.5 m) and flying speed (3 to 5 m/s) on deposition uniformity and reported that application of pesticide at a flying height of 1.5 m and a flying speed of 5 m/s observed more uniform droplet deposition than the conventional sprayer on rice crops. At a constant flying height of 2 m, with the increase of flow rate, the droplet density and coverage also increased. However, at a constant flow rate (1.08 L/min), with the increase of flight height and flying speed, the droplet density decreased (Wang et al., 2017). The UAV can apply chemicals precisely and accurately on the target area by flying and hovering close to the plant canopy of crops. Ahmad et al. (2020) reported that the operational parameters of UAV achieved the highest chemical deposition on weed canopy at a flight height and a flying speed of 2 m and 2 m/s, respectively. Paul et al. (2023) studied the efficacy of herbicides on rice crops and reported that the efficacy of UAVs and backpack sprayers has no significant difference. Similarly, Pranaswi et al. (2024) showed that the efficacy of systematic herbicide on wheat was not influenced by UAV or backpack application. Gao et al. (2024) reported that the droplet deposition characteristics of UAV were better at a flight height, a flying speed, and a spray volume of 1.5 m, 2 m/s, and 180 L/hm^2^, respectively. The droplet distribution was improved by lowering flight height and flying speed and increasing spray volume. Reducing flight height and flying speed can enhance the distribution of droplets in canopy (24, 25, 26). Kharim et al. (2019) investigated the droplet deposition of liquid fertilizer on rice crops using UAV. They reported that the lower flying speed (2 m/s) had greater droplet deposition per unit time as well as higher droplet uniformity as compared to higher flying speeds (4 and 6 m/s). Chen et al. (2020) conducted an experiment using a multi-rotor UAV and four TEEJET nozzles with different droplet sizes and the same spraying rate on a rice crop. The result showed that the droplet size significantly influenced the deposition of droplets and drift.

Pesticides persist as the most common way to control thrips. Thrips are mostly controlled by pesticides including organophosphorus, carbamate, pyrethroid, and neonicotinoid. Different insecticides have distinct modes of action, and their efficiency in thrip control differs to a certain extent. The systematic neonicotinoid insecticides are absorbed by plant roots or leaves and distributed throughout the entire plant canopy. Thrips absorb these systemic neonicotinoid insecticides when they penetrate plant tissues and suck sap. The acetylcholine, a neurotransmitter involved in nerve signal transmission, is mimicked by the pesticides. The persistent nerve signal transmission results from its irreversible binding to the nAChRs in thrips nerve cells. Acetylcholinesterase is unable to break down neonicotinoids, which causes nerve signals to continue firing erratically. This leads to hyperexcitation, which causes tremors, muscle contractions, and loss of coordination. Ultimately, overstimulated nervous systems fail and cause thrips to be paralyzed and finally die. Excessive use of pesticides has led to increased insecticide resistance in thrips (Huseth et al., 2016). Using natural enemies**’** predatory functions to manage thrips has also become an important strategy. Zhang et al. (2020) studied the above method, which improves pesticide deposition on crops, reduces mechanical damage, and increases operational efficiency as compared to conventional sprayers. Thrips are difficult to avoid and control because of their short growth period, high reproductive capacity, rapid outbreaks, and considerable generation overlap. Chen et al. (2016) showed that lotus thrips (*Scirtothrips dorsalis Hood*) can be effectively monitored by applying avermectin by UAVs. Yuan et al. (2017) reported that UAV spraying produced greater control effects on cowpea (*Vigna unguiculata L.*) thrips [*Megalurothrips usitatus* (Bagnall)] than manual spraying at lower dosages. Lin et al. (2020) showed that the use of UAVs to spray pesticides achieved an 83.5% control on sugarcane thrips. The recommended amount resulted in a higher thrips control effect than manual spraying, and the required dosage was 25% lower (Liu et al., 2023). Fang et al. (2023) carried out field studies to evaluate the droplet density, coverage rate, deposition amount, droplet uniformity, and control effect on cotton thrips in the cotton canopy after spraying 25% thiamethoxam water-dispersible granules via UAV, and their results found the optimal parameters for using a UAV to control thrips during flowering in cotton fields. The thrips controlled was: 80.51% and 79.22% for 10% cyantraniliprole OD and 10% spinetoram SC after 7 days of spraying by UAV, respectively.

The spray characteristics of each UAV model differ from others in terms of their platform design, payload, nozzle types, combinations, etc (Hunter et al., 2020; Sinha et al., 2022). The variation between two UAV models, assessing and optimizing operational parameters for effective spray deposition and uniformity, is difficult, particularly under changing climatic variability (He, 2018). Lan et al. (2017) conducted an experiment on five commercially available UAVs and reported that each model has its unique optimal condition for maximum spray deposition. Similarly, Martin et al. (2019) and Sinha et al. (2022) found that the spray characteristics vary at the same operational parameters for two different UAV models. To enable such precision applications, comprehensive evaluation and understanding of the spray performance (deposition, uniformity, and drift) of various UAAS is essential, which will determine optimal management strategies for their effective use. Furthermore, UAAS design and functionality are continuously evolving on emerging platforms, as well as between various models from the same manufacturer, due to differences in physical and operational characteristics. To apply information from one platform to another, it is important to assess spray performance in various operational circumstances to determine the most effective application parameters.

Based on the above literature, there is a lack of thorough investigation of operational parameters (flight height and flying speed) of UAVs on performance parameters such as droplet density, droplet size, coverage, spray deposition, relative span factor, and drift characteristics on each canopy zone of the pigeon pea crop. The commercially available UAV manufacturers are adopting UAVs before analyzing their spray performance, including flight height, flying speed, flow rate, type of nozzle, arrangement of nozzle, spray uniformity, and application rates. The present study was taken up to study the effect of different flight heights and flying speeds on spray performance parameters such as droplet density, droplet size, coverage, spray deposition, and drift characteristics of UAV. These data are required to enhance the UAV spraying techniques to late-season spraying areas for growers, farmers, manufacturers, extension workers, researchers, and UAV operators. Therefore, this study was conducted to (1) investigate the effect of operational parameters of UAV for efficient agrochemical application in pigeon pea crop and (2) control thrips on pigeon crop at different flight heights and flying speeds. The specific objective was to measure spray droplet deposition density, droplet size, spray coverage, spray deposition, relative span factor, and drift characteristics at three flying heights (1.5, 2, and 2.5 m above crop canopy) and flying speeds (2, 2.5, and 3 m/s).

## 2 Materials and methods

### 2.1 Experimental site

**Figure 1:**
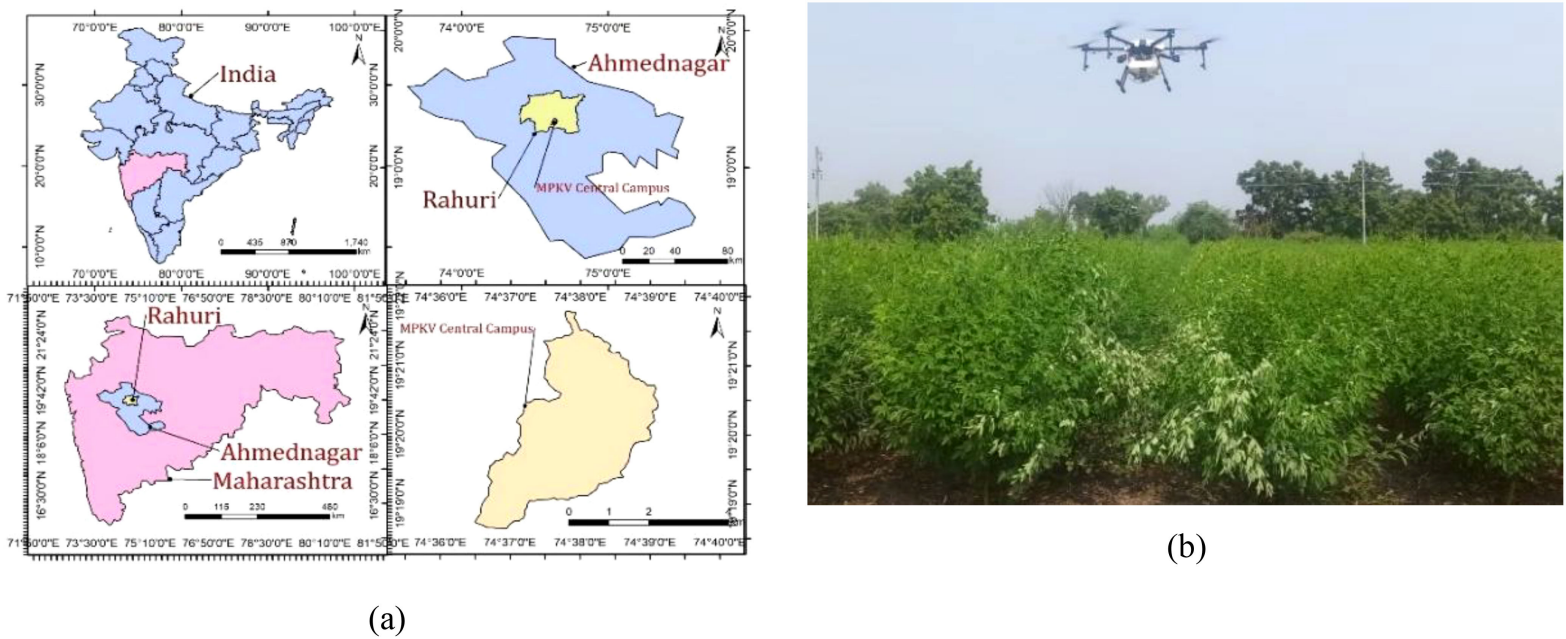
**(a)** Experimental site selected for the evaluation of the UAV-based spraying system and **(b)** spraying operation in the pigeon pea field.

The experiment was carried out in a pigeon pea field (**Figure 1**, 19.3926°N, 74.6488°E) in the research farm of Mahatma Phule Krishi Vidyapeeth, Rahuri, Maharashtra, India, from 09:30 to 11:00 and 16:00 to 18:00 in October 2023. Various crop parameters, such as crop height, plant width, and row-to row-distance, were measured during each experimental field of UAV. A detailed description of the experimental field and the canopy attributes of the pigeon pea crop is provided in **Table 1**.

**Table 1:** Details of the experimental field plot and canopy attributes of the pigeon pea crop.

Parameter	Value
Variety	Phule Trupti
Crop growth stage	Flowering
Crop type (rainfed/irrigated)	Irrigated
Plot size, m × m	22 × 10
Row spacing, m	0.9
Plant spacing, m	0.9
Plant height, m (mean ± SD)	2 ± 0.06
Plant width, m (mean ± SD)	0.85 ± 0.04
Date of sowing	June 2023

### 2.2 UAV spray system

**Figure 2:**
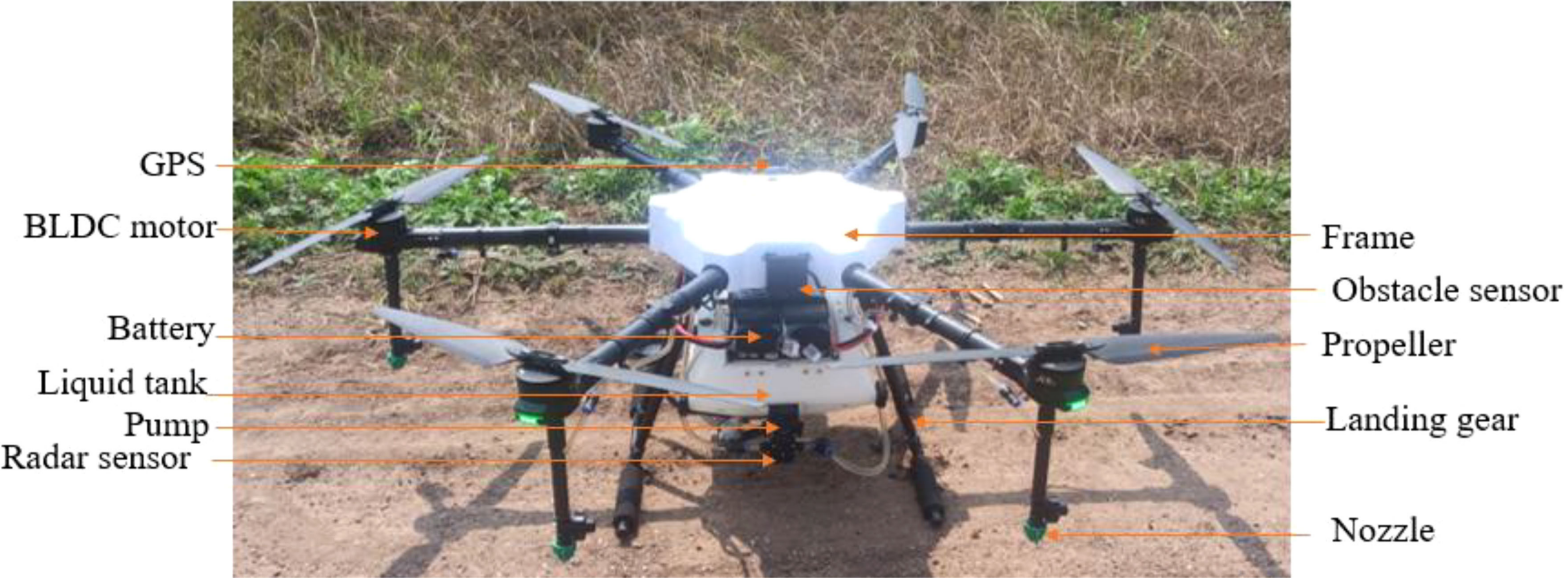
Battery-operated hexacopter UAV.

A six-propeller battery-operated hexacopter UAV sprayer (Agribot, Iotechworld, Gurugram, Haryana, India) was used in the spraying experiment (**Figure 2**). It consists of a main frame, a propeller, a BLDC motor, a liquid tank, a water pump, a lithium polymer (LiPo) battery, a flight controller, an RC receiver, four GNSS units, and a high-pressure flat spray nozzle. The UAV sprayer has two LiPo batteries each of six cells with a capacity of 16,000 mAh to supply the necessary current required for the propulsion system. A BLDC motor (24 V) was used with a pump to pressurize spray liquid and then to atomize it into fine spray droplets. This UAV has four flat fan nozzles that were mounted exactly below the propeller. Exactly below the liquid tank, a radar-based collision avoidance and terrain following sensor was used to maintain the height of spraying according to crop height. Also, an obstacle sensor was placed on the front side mounted on the main frame, which indicates any obstacle, such as transmission lines and poles, to adjust the height of spraying. This UAV model has the function of GPS route planning and RTL, which can complete its spraying operations autonomously. The detailed specification of the hexacopter UAV is given in **Table 2**.

**Table 2:** Specification of the hexacopter UAV.

S. no.	Name of the item	Value
1	Frame configuration	Hexacopter
2	Folded size (L × B × H), mm	762 × 762 × 483
3	Maximum take-off weight, kg	23.2
4	Range of Operation, km	5
5	Endurance in working, min	20
6	Tolerable wind speed, m/s	10 to 12
7	Payload, kg	10
8	Flight modes	Fully autonomous, semi-autonomous, and manual
9	Spray tank, L	10
10	Flight height, m	1 to 10
11	Flying speed, m/s	1 to 8
12	Swath width, m	2 to 4 m
13	Type of nozzle	High pressure flat spray
14	Number of nozzles	4
15	Discharge rate per nozzle, L/m	0.850
16	Liquid pressure, kg/cm^2^	3.2
17	Charging time, min	30–40

### 2.3 Calibration of UAV and flow rate

The UAV was calibrated to determine the amount of water required for a 1-ha area. A UAV tank was filled with a known amount of water and sprayed on the marked field. The start point and end points were marked with poles, and the sprayed area was taken into account. An amount of water required per hectare was determined with Equation 1 (Yallappa et al., 2024).


(1) $$\begin{array}{c} Volume\;of\;watar\;required,\;(l/ha)\\ =\;\frac{\left\{volume\;of\;water\;used\;(L)\times 10000\right\}}{Test\;area} \end{array}$$

After the calibration of UAV, the water requeirment per hecatre was 45 L/ha with the same flow rate of 0.850 L/min for each nozzle.

### 2.4 Application rate

The application rates were measured by using the American Society of Agricultural and Biological Engineers standard (ASABE) (ASABE Standards, 2018). The actual water required per hectare was determined by Equation 2.


(2) $$\text{Application rate }(\text{R})=\;\frac{\text{Q}\times \text{K}}{\text{S }\times \text{W}}$$

where,

R = Application rate, L.ha^−1^

Q = Output rate, L.min^−1^

K = Constant, 600

S = Flying speed, km.h^−1^

W = Effective swath width, m

### 2.5 Experimental design

A systemic neonicotinoid insecticide is a systematic insecticide used for various diseases or pests in the pigeon pea crop including thrips. A mixture of systemic neonicotinoid insecticide and water (1 mL of systemic neonicotinoid insecticide per liter of water) was used as a spraying solution during spraying on the pigeon pea crop. The randomized block design (RBD) was used for the experiment with three flight heights (1.5, 2, and 2.5 m) and flying speeds (2, 2.5, and 3 m/s) to measure dependent parameters on the top, middle, and bottom canopy of the pigeon pea crop. **Table 3** shows a treatment combination of two factors (2) each having three levels (3) with three replications (3). The droplet deposited in a unit area, droplet size, coverage, spray deposition, uniformity coefficient, and relative span were observed. The meteorological parameters during spraying were taken from the Department of Meteorology, MPKV, Rahuri. The meteorological parameters, i.e., temperature, wind speed, relative humidity, and gust speed, were 23.66 to 26.40°C, 1.95 to 2.35 m/s, 57.65% to 65.20% and 1.82 to 2.17 m/s, respectively, during the experiment (**Table 4**). During the spray trials, the weather parameters (**Table 4**) were within the recommended limits for spray applications (Sahni et al., 2022; Martin et al., 2019; Tepper, 2017).

**Table 3:** Test factors and levels.

Independent parameters	Levels	Replicate	Dependent parameters
Flight height	3 (1.5, 2, and 2.5 m above plant canopy)	3	Coverage (%) Spray deposition, µl/cm^2^ Droplet density (*n*/cm^2^) Droplet size (µm) Relative span factor
Flying speed	3 (2.0, 2.5, and 3.0 m/s)		
Canopy zone	3 (top, middle, and bottom)		

**Table 4:** Meteorological parameters measured during each treatment of spraying operation of UAV.

Treat	Flight height, m	Flying speed, m s^−1^	Time of test	Temp, °C	Wind speed, m s^−1^	Relative humidity, %	Gust speed, m s^−1^	Wind direction
T1	1.5	2.0	09:30 to 09:45	24.35	1.90	57.61	1.82	W-E
T2	1.5	2.5	09:46 to 10:00	24.31	2.10	58.58	2.05	W-E
T3	1.5	3.0	10:01 to 10:15	24.85	2.10	60.25	1.92	W-E
T4	2.0	2.0	10:16 to 10:30	25.21	2.20	61.48	1.99	W-E
T5	2.0	2.5	10:31 to 10.45	25.53	2.35	61.90	2.14	W-E
T6	2.0	3.0	10.46 to 11:00	25.80	2.32	63.35	2.17	W-E
T7	2.5	2.0	16:00 to 16:15	26.00	2.19	64.38	1.86	W-E
T8	2.5	2.5	16:16 to 16:30	26.40	2.21	65.20	2.78	W-E
T9	2.5	3.0	16:31 to 16:45	25.25	2.22	62.10	2.04	W-E

### 2.6 Layout of water-sensitive paper (WSP) placement

**Figure 3:**
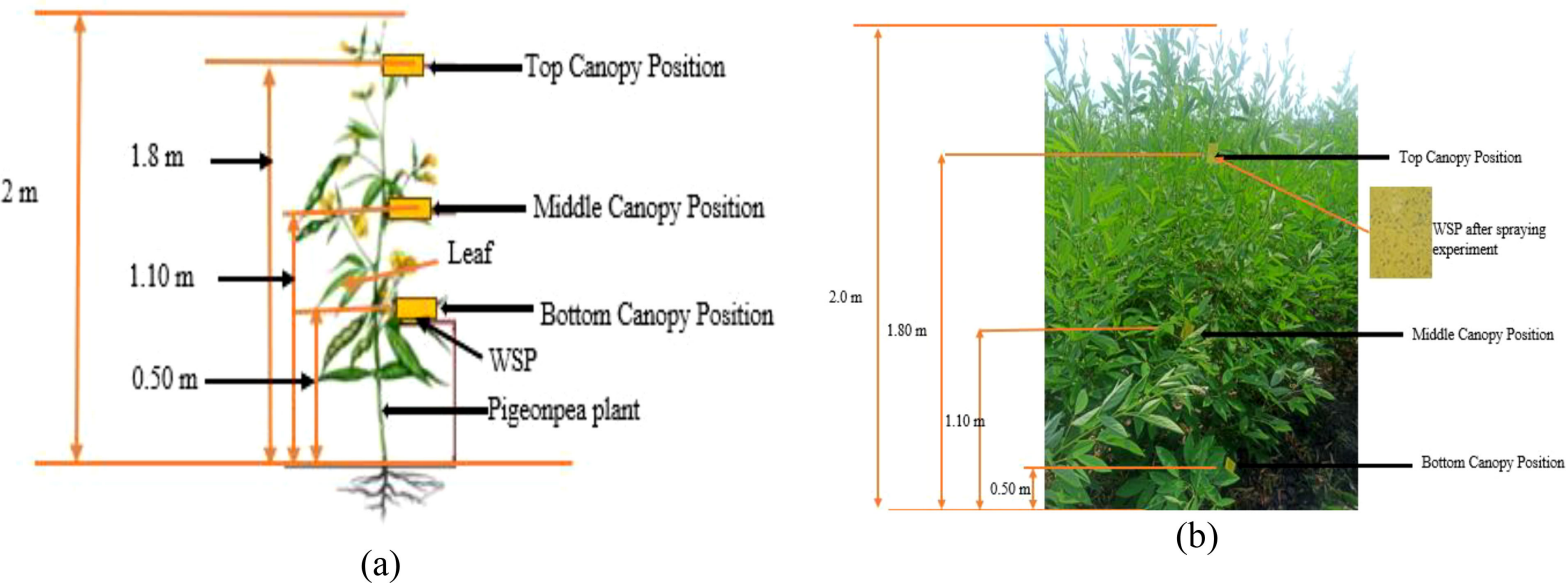
**(a)** Layout of water-sensitive paper (WSP) placement on the leaves and **(b)** the position of WSP for the collection of various performance parameters.

Water-sensitive paper (WSP) cards (76 × 50 mm, Syngenta, AG, Basel, Switzerland) were used to quantify the deposition of droplet density (Yallappa et al., 2022). The WSP was attached vertically at three leaves of the pigeon pea crop on the bottom, middle, and top at 0.5, 1.10, and 1.80 m from the ground, respectively (**Figure 3**).

### 2.7 Sampling site design for drift calculation

**Figure 4:**
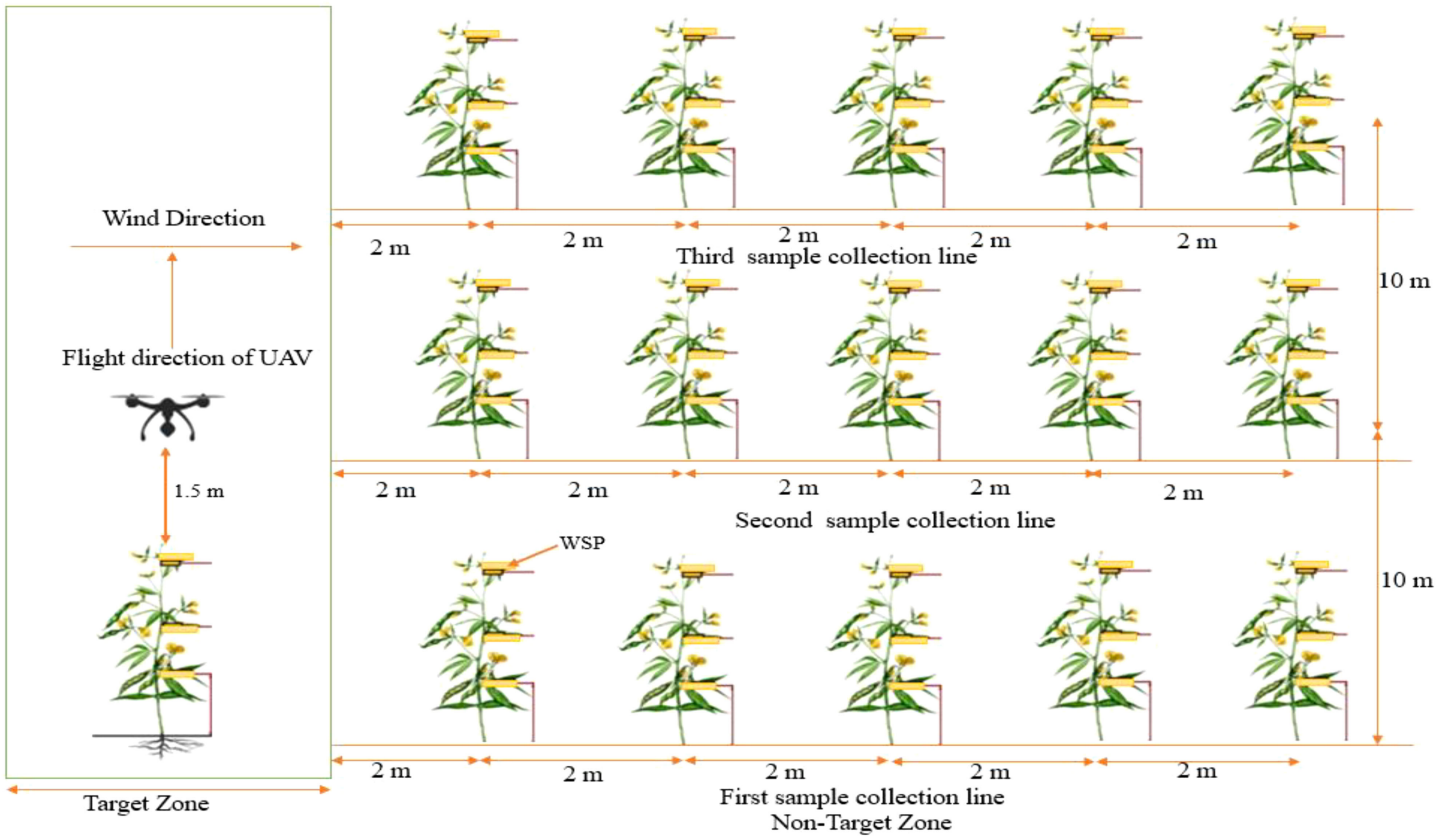
Sampling layout for UAV performance evaluation.

The experimental field was divided into two distinct zones, i.e., the target zone and the off-target zone. The actual target zone is 4 m, while after the actual target zone, the end point (right side) to 10 m was considered as the off-target zone. To measure spray drift, only the right side (off-target zone) was selected because no droplet deposition observed on the left side of the target zone. For sampling, three collection lines were set up, each 10 m wide perpendicular to the direction of drone flight (**Figure 4**). The WSPs were attached vertically to the bottom, middle, and top canopies of the pigeon pea crop (0.5, 1.10, and 1.80 m, respectively). To quantify spray drift, the WSPs were placed every 2 m, beginning from the target zone and increasing in distance to 2, 4, 6, 8, and 10 m (Yallappa et al., 2022). The experimental field layout is shown in **Figure 4**. To assess the droplet penetration into the pigeon pea canopy, samples were collected from the target zone. Moreover, spray drift was quantified using samples from 2 to 10 m (every 2 m apart) from the off-target zone. To collect and analyze spray droplet parameters, 54 sapling cards were used for each UAV run.

### 2.8 Performance parameters

#### 2.8.1 Droplet density (deposits/cm^2^)

This is the number of spray droplets deposited per unit surface area on WSP (Yallappa et al., 2022).

#### 2.8.2 Spray coverage (%)

This is the ratio of droplets covering the target surface region to the total target surface region (Qi et al., 2020).

#### 2.8.3 Volume mean diameter

Volume mean diameter, or VMD, is the average drop size that is obtained when the volume of smaller drops consists of 50% of the liquid that is sprayed through the nozzle; half of the volume is atomized into smaller droplets, while the other half is larger than the VMD (Srinivasarao et al., 2021).

#### 2.8.4 Number median diameter

A droplet with a size such that half of the total number of droplets are smaller than it and half are larger is used to indicate the number’s median diameter (Srinivasarao et al., 2021).

#### 2.8.5 Relative span factor

The relative span factor decreases the spray spectrum to one number, which confirms the uniformity of droplet size distribution. A uniform spray was achieved when the relative span factor values were close to one. The relative span factor was calculated using Equation 3 (Srinivasarao et al., 2021).


(3) $$\text{Relative span factor}=\frac{\left|{\text{D}}_{\text{v}0.9}-{\text{D}}_{\text{v}0.1}\right|}{{\text{D}}_{\text{v}0.5}}$$

where,

D_v0.9_ = 90% of the total volume of the sample’s droplets

D_v0.5_ = 50% of the total volume of the sample’s droplets

D_v0.1_ = 10% of the total volume of the sample’s droplets

#### 2.8.6 The coefficient of variation (%)

This represents the deposition of droplets and the uniformity of coverage parameters on the plant canopy (Qi et al., 2023). The coefficient of variation (CV) and standard deviation (SD) of sample was calculated by following Equations 4 and 5, respectively.


(4) $$\text{CV }(\%)=\frac{\text{SD}}{\bar{\text{X}}}\times 100$$

where,

SD = standard deviation of the sample; 

$\bar{\text{X}}$ = average coverage parameters of the droplets.


$$\bar{\text{X}}=\;\frac{\sum {\text{x}}_{\text{i}}}{\text{N}}$$

(5) $$\text{SD}=\;\sqrt{\frac{\sum_{\text{i}=1}^{\text{n}}{\left({\text{X}}_{\text{i}}-\bar{\text{X}}\right)}^{2}}{\left(\text{n}-1\right)}}$$

where,

X*_i_* = droplet coverage of each sampling point

n = number of sampling points of each test group

### 2.9 Collecting and analyzing spray droplet data

#### 2.9.1 Collection of WSP

The WSP was allowed to dry after every test before being collected into labeled zip-lock bags and stored in a dry place to ensure that the WSP cards were transferred into the lab safely. During the collection of WSP cards, wearing hand gloves is essential to avoid moisture transfer. The various performance parameters were determined on the top, middle, and bottom canopy as suggested by Yallappa et al. (2022).

#### 2.9.2 Analysis of WSP to determine spraying effectivity

**Figure 5:**
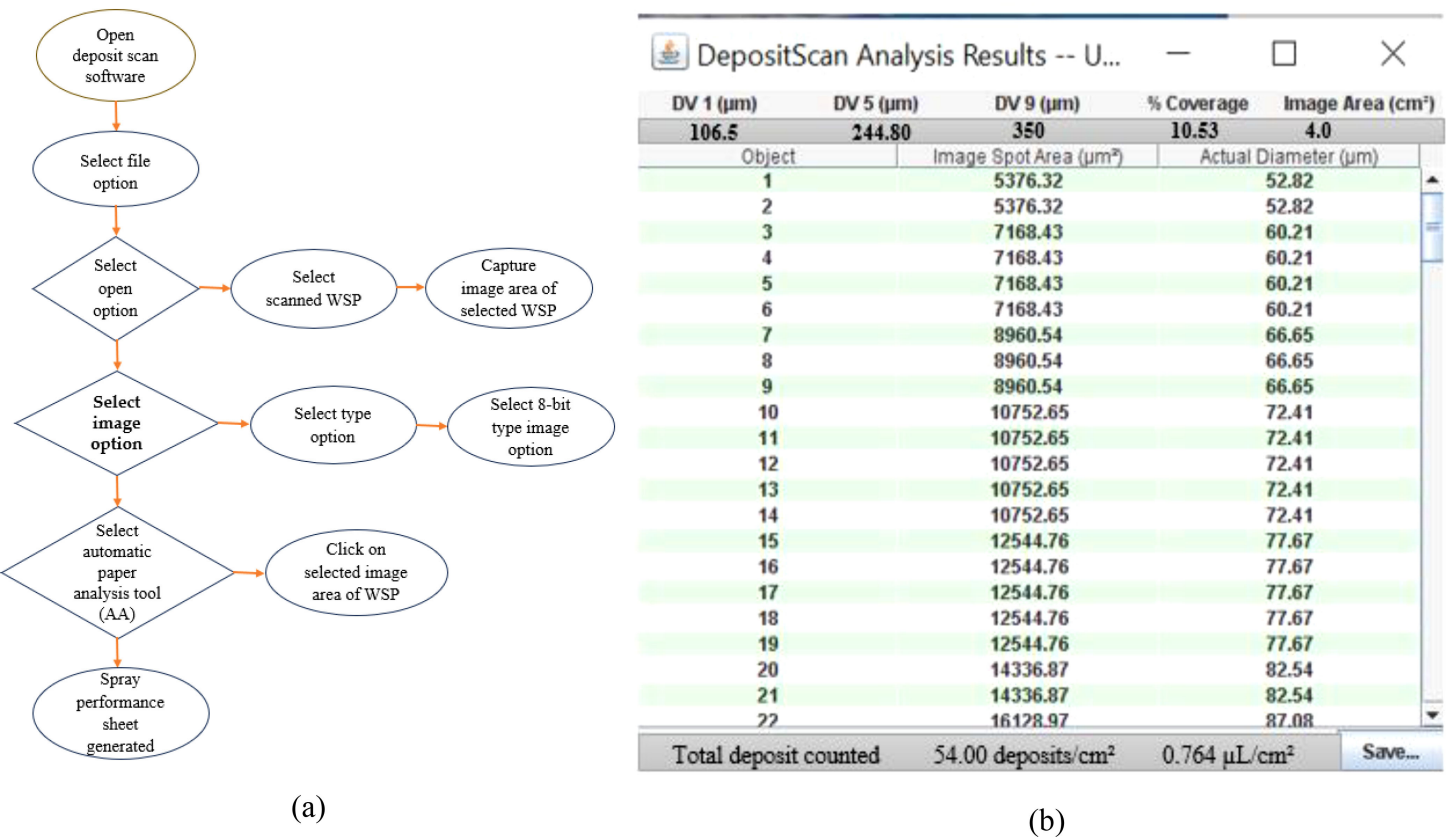
**(a)** Step-by-step procedure for WSP analysis and **(b)** spray performance parameter sheet via the Deposit Scan Software.

WSPs were scanned in 600-dpi images by using a high-density lab scanner. After WSP scanning, the Deposit Scan Software (ImageJ 1.38x, USDA, Wooster, OH, USA) was used to extract the spray characteristics data, i.e., volume mean diameter (µm), number median diameter (*n*), number of droplets per unit area (*n*/cm^2^), spray coverage (%), and spray deposition (µl/cm^2^) (47, 48). The various performance parameters were determined on the top, middle, and bottom canopy suggested in (43). **Figure 5a** depicts a step-by-step procedure for WSP analysis via the Deposit Scan Software (**Figure 5b**).

### 2.10 Control of thrips

A systemic neonicotinoid insecticide is a contact herbicide used to control thrips on pigeon pea crops. A systemic neonicotinoid insecticide mixture (1 mL of systemic neonicotinoid insecticide per liter of water) was used as a sprayer solution for thrips control after 1, 5, and 10 days of spraying experiment.

A thrips population growth assessment was conducted during the pigeon pea flowering time (1–10 October 2023). Monitoring was carried out at intervals of 2 days, with a regular survey time of 08:00 to 17:00. Five-point sampling was used to determine the number of thrips in the survey area (14 m × 10 m). Ten pigeon pea leaves were randomly selected. The thrips were removed from the leaf and placed in yellow sticky card traps. The number of thrips at each instar was measured.

A study of thrips activity patterns throughout the pigeon pea flowering period was carried out on 15 October 2023 (with a mean of more than 1,000 thrips per 50 pigeon pea plants). An experiment was carried out every 2 days. Yellow sticky card traps (attached to a 1-m-long pole) were installed at five different locations in the sampling region (14 m × 10 m) to assess the number of active thrips outside the leafs. The amount of thrips on pigeon pea leafs was determined using five-point sampling, with 50 pigeon pea leavess picked at random from the same study test and replicated three times. Each test was carried out every 2 h between 08:00 and 17:00 and was repeated three times.

Thrips counts have been measured and counted according to pesticide field efficiency testing guidelines. A parallel jump method has been adopted to measure the total number of thrips in a five-point survey using a single pigeon pea leaf per point to determine the control effect of the applied pesticide on thrips. The total control effect of thrips was measured without considering the different growth stages of thrips. The dropping rate and control effect of thrips in each test were calculated by counting the total numbers of live insects before and after spraying operations using the Equations 6, 7 (Lou et al., 2018).


(6) $$D=\;((N_{a}-N_{b})/N_{a})\;\times 100$$

(7) $$CE=\;(D_{a}-D_{b})/(100-D_{b})\;\times \;100$$

where,

D = Decline rate of insect mouth.

N_a_ = Total number of live insects before spraying.

N_b_ = Total numbers of live insects after spraying.

CE = Control efficiency, %.

D_a_ = Decline rate of insect mouth in the treatment area.

D_b_ = Decline rate of insect mouth in the control area.

### 2.11 Statistical analysis

Analysis of variance (ANOVA) was performed by using a three-way RBD to determine if there are significant differences in spray performance parameters at different flight heights and flying speeds on each canopy of the pigeon pea crop. The experiment was replicated three times, and statistical analysis was carried out in OPSTAT software (O. P. Sheoran, a computer programmer at CCS HAU, Hisar, India) to determine the level of significance at 5%.

## 3 Results and discussion

### 3.1 Spray coverage

**Figure 6:**
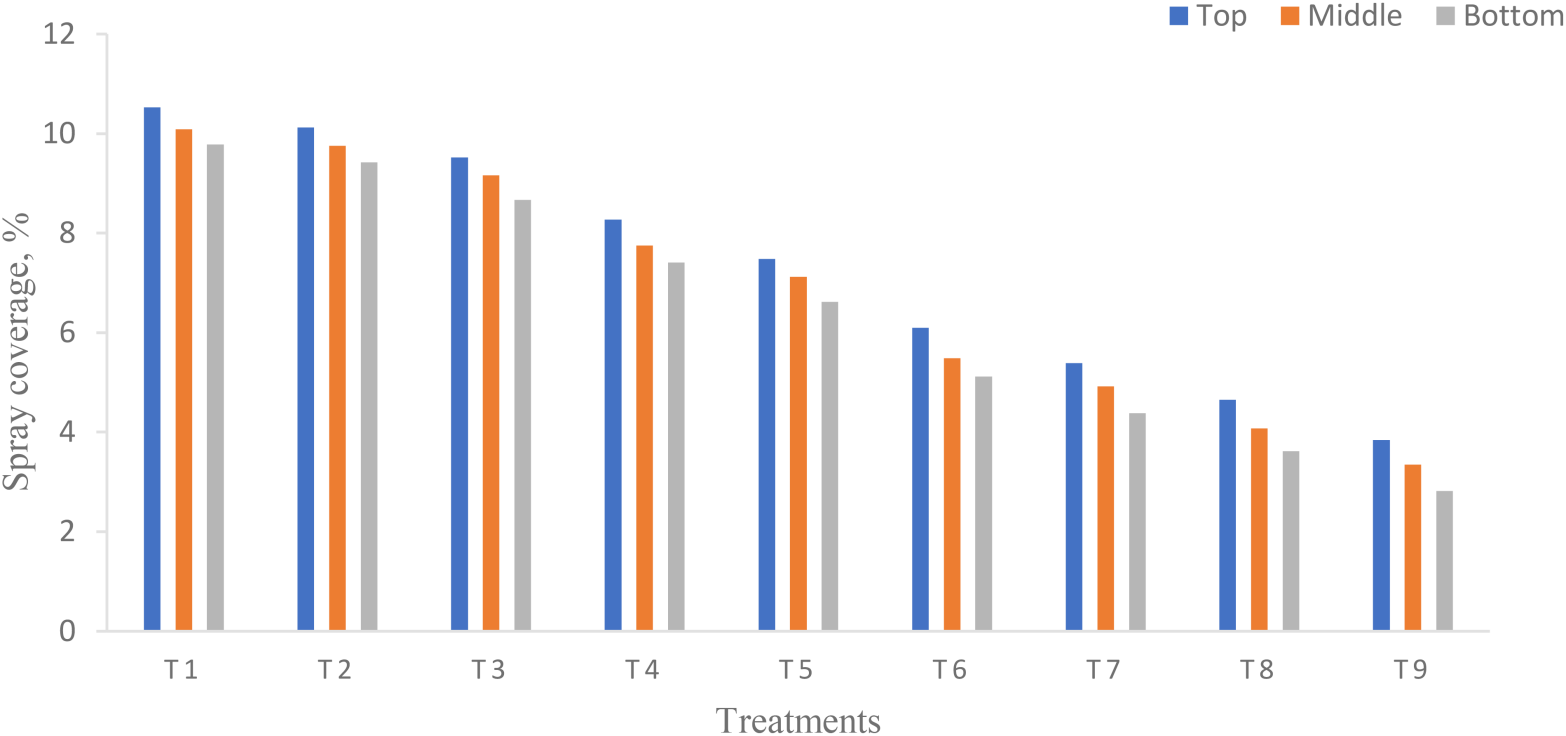
Effect of flight height and flying speed on coverage at different canopy positions.

ANOVA (**Table 5**) shows that there was a significant difference in spray coverage on each canopy of pigeon pea crop at different flight heights and flying speeds and their interaction (*p* < 0.05). From **Figure 6**, the highest spray coverage was observed on the top canopy as compared to the middle and bottom canopy on the pigeon pea crop in the target zone. The highest and lowest spray coverage for treatments T_1_ and T_9_ are 10.53% and 3.84% on the top, 10.09% and 3.35% on the middle, and 9.78% and 2.82% on the bottom canopy of pigeon pea crop, respectively. Similarly, Vitoria et al. (2023) reported that the maximum coverage on the top, middle, and bottom canopy was 6.98%, 4.50%, and 2.21% at a flight height of 2.5 m above the crop canopy and at a flying speed of 5 m/s. Also, a significant difference was observed with an increase in flight height from 1 to 3 m (Chen et al., 2021). The coverage on the coffee plantation on the top and bottom canopy was 10.5% and 4.8% at a flight height above the ground surface of 3 and 4 m, respectively (Souza et al., 2022). In addition, the coverage was found to be higher on the top canopy of plants as compared to the middle and bottom canopy in the target zone (central line) because of increased flight height and flying speed there is decrease in deposition of droplets. The reason may be due to increased flight height and flying speed scatters the droplets away from target zone which reduces coverage as well as wind deviates the droplets from target zones. There was a higher coefficient of determination (*R* ^2^) of 0.9990 for the pigeon pea plant, which indicates that the 99.90% variability of the response could be explained, which is statistically accurate (Askari et al., 2021).

**Table 5:** Analysis of variance of coverage data at different flight heights and flying speeds.

Sources	DF	Sum of squares	Mean squares	*F*-calculated	*p*-value
Replication	2	0.02			
Flight height, m	2	41.31	207.16	143.52	0.0000*
Flying speed, m/s	2	35.51	17.75	12.45	0.0000*
Canopy position	2	10.65	5.32	36.45	0.0000*
Flight height × Flying speed	4	3.87	0.96	66.27	0.0000*
Flight height × Canopy position	4	0.20	0.04	29.38	0.0000*
Flying speed × Canopy position	4	0.02	0.004	2.52	0.0495*
Flight height × Flying speed × Canopy position	8	0.03	0.004	2.55	0.019*
error	52	0.07	0.001		
Total	81	91.68			
CD@5%: 0.0627, CV = 4.04, *R* ^2^ = 0.9990					

CV, coefficient of variation, *p < 0.05: significant at 5% level of significance; NS, non-significant.

Moreover, the smaller the coefficient of variation (CV), the better the coverage (Yao et al., 2017). The CV in the target zone (central line) was 4.04 while CD was 0.9990. To achieve maximum application efficiency, agrochemicals must be applied to the entire plant canopy, including the top, middle, and bottom canopy of plants. The spray coverage on the bottom portion of the plants is lower because of the obstruction presented by the top leaf mass of the pigeon pea. The WSP cards exhibited this phenomenon. Spraying is considered as overspraying when the coverage is more than 30% (Biglia et al., 2020).

### 3.2 Spray deposition

**Figure 7:**
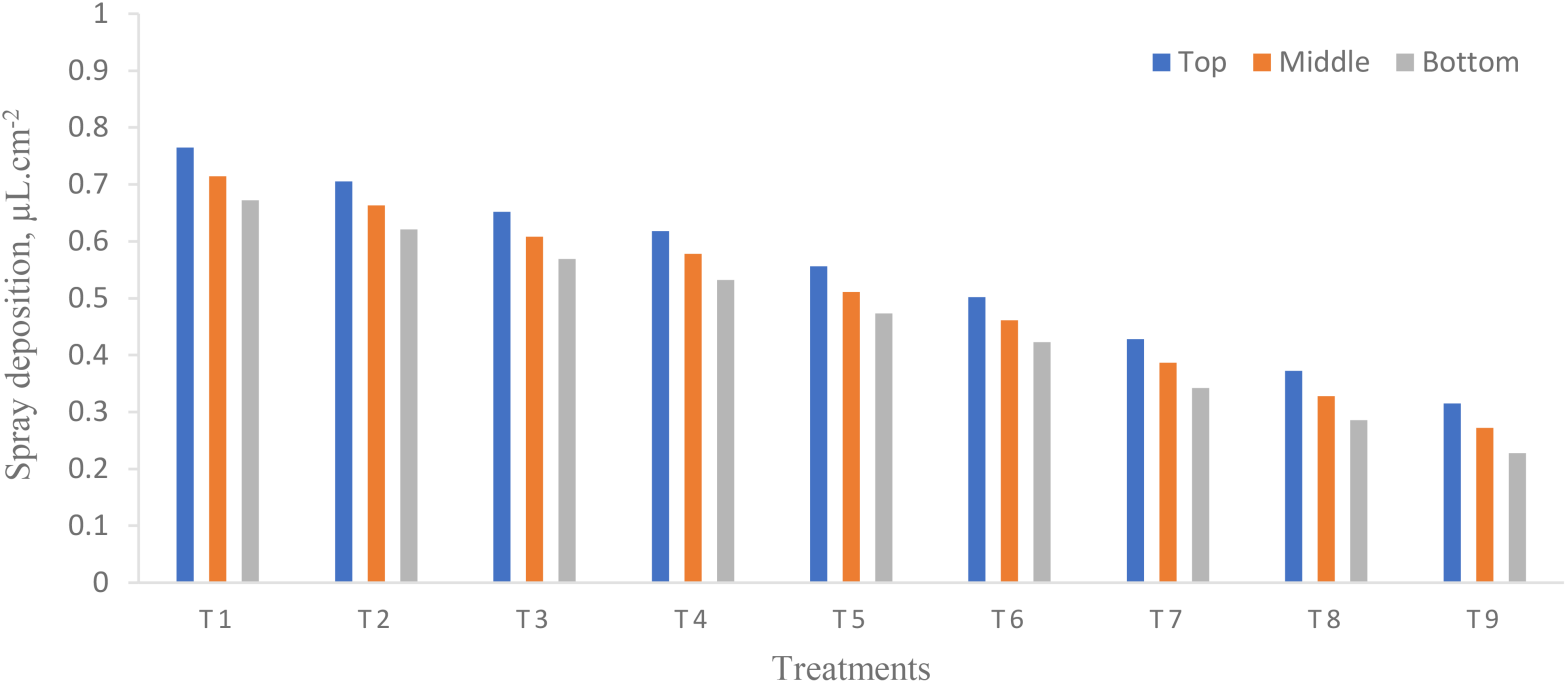
Effect of flight height and flying speed on spray deposition at different canopy positions.

Spray deposition is an essential aspect that has a direct impact on the effectiveness of UAV sprays. ANOVA (**Table 6**) shows that there was significant difference in spray deposition on each canopy of pigeon pea crop at different flight heights and flying speeds except their interaction (*p* < 0.05). From **Figure 7**, the highest spray deposition was observed on the top canopy as compared to the middle and bottom canopy on the pigeon pea crop in the target zone. The highest and lowest spray coverage for treatments T_1_ and T_9_ were 0.764 and 0.313 µl/cm^2^ on the top, 0.714 and 0.272 µl/cm^2^ on the middle, and 0.672 and 0.225 µl/cm^2^ on the bottom canopy of the pigeon pea crop, respectively. There was a significant difference in spray deposition at the central line along with the observation line. Generally, there is a strong relation between coverage and spray deposition rate. The maximum spray deposition was 0.02 µl/cm^2^ at 200 µm and 0.01 µl/cm^2^ at 100 µm (Chen et al., 2021). The highest and lowest spray deposition on the top canopy on Areca Catechu was 0.16 and 0.03 µl/cm^2^ at a flight height and a flying speed of 8.84 m and 1.5 m/s, and 10.31 m and 2.5 m/s, respectively (Wang et al., 2023a). The higher spray deposition on the top, middle, and bottom canopy was 0.61, 0.25, and 0.12 µl/cm^2^ at a flight height and a flying speed of 2.5 m and 5 m/s, respectively (Vitoria et al., 2023).

**Table 6:** Analysis of variance of spray deposition at different flight heights and flying speeds.

Sources	DF	Sum of squares	Mean squares	*F*-calculated	*p*-value
Replication	2	0.000			
Flight height, m	2	1.538	0.769	34.73	0.00000*
Flying speed, m/s	2	0.171	0.085	38.60	0.00000*
Canopy position	2	0.096	0.048	21.68	0.00000*
Flight height × Flying speed	4	0.001	0.000	5.68	0.00072*
Flight height × Canopy position	4	0.000	0.000	0.75	0.56089NS
Flying speed × Canopy position	4	0.000	0.000	0.03	0.99785NS
Flight height × Flying speed × Canopy position	8	0.000	0.000	1.50	0.17804NS
error	52	0.001	0.000		
Total	81	1.807			
CD@5%: 0.0077, CV = 6.77, *R* ^2^ = 0.9992					

CV, coefficient of variation, *p < 0.05: significant at 5% level of significance; NS, non-significant.

Moreover, the highest spray deposition was observed on the top canopy as compared to the middle and bottom canopy because increased flight heights and flying speeds decreased the deposition of droplet density, which leads to lowering spray deposition. Similarly, Ahmad et al. (2020) reported that the increase in flight height and flying speed decreased spray deposition. From **Figure 7**, it was found that the flight height and flying speed had a negative effect on spray deposition. Similar results have been reported by various researchers (Chen et al., 2021; Martin et al., 2019; Wang et al., 2023b). There was a higher *R* ^2^ of 0.9992 for the pigeon pea crop, which indicates that the 99.92% variability of the response could be explained, which is statistically accurate (Askari et al., 2021).

**Figure 8:**
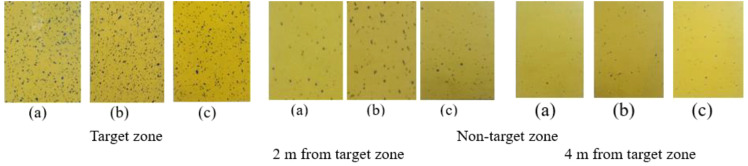
Collected representative WSP from target and non-target zones after spraying operation from top **(a)**, middle **(b)** and bottom **(c)** canopy zone of pigeon crop.

Moreover, the smaller the CV, the better the coverage (Yao et al., 2017). The CV in the target zone (central line) was 6.77 while CD was 0.9972. To achieve maximum application efficiency, agrochemicals must be applied to the entire plant canopy, including the top, middle, and bottom canopy of plants. The spray deposition on the bottom portion of the plants is lower because of the obstruction presented by the top leaf mass of the pigeon pea. The WSP cards exhibit this phenomenon (**Figure 8**).

### 3.3 Droplet density

**Figure 9:**
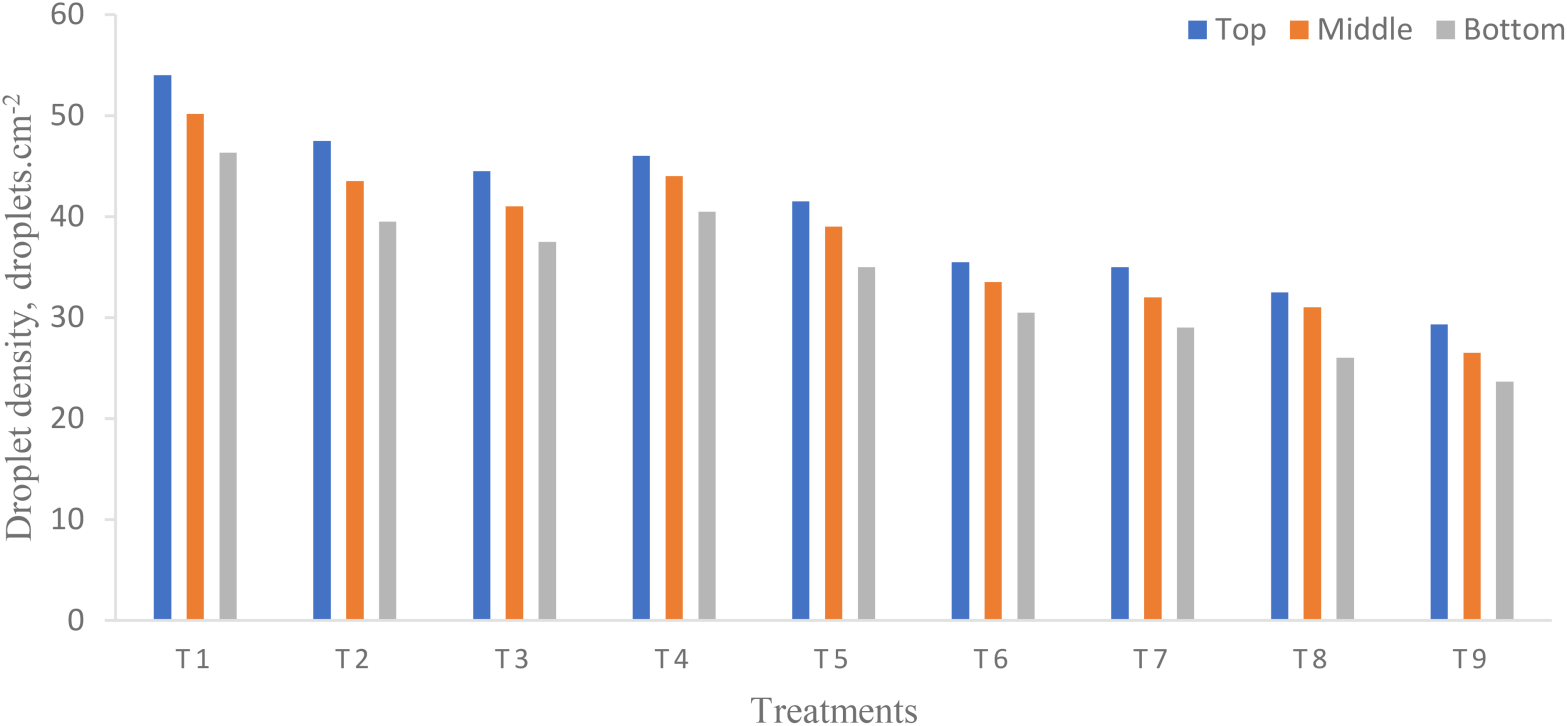
Effect of flight height and flying speed on droplet density at different canopy positions.

In the present study, droplet density is considered as one of the most important performance parameters to evaluate the effect of flight height and flying speed of UAV because it directly affects the spray deposition rate and coverage. ANOVA (**Table 7**) shows that there was a significant difference in droplet density on each canopy of pigeon pea crop at different flight heights and flying speeds except their interaction (*p* < 0.05). From **Figure 9**, the highest droplet density was observed on the top canopy as compared to the middle and bottom canopy on the pigeon pea crop in the target zone. The highest and lowest droplet density deposited for treatments T_1_ and T_9_ were 54.00 and 29.33 droplets/cm^2^ on the top, 50.17 and 26.50 droplets/cm^2^ on the middle, and 46.33 and 23.67 droplets/cm^2^ on the bottom canopy of the pigeon pea crop, respectively. Overall, the maximum droplet density deposited on the top canopy as compared to the middle and bottom canopy zones because of the strong downwash produced by the UAV propeller was due to the lowered flight height; an overall interactive influence between the horizontal and vertical airflow-led droplets breaks up and scatters from the target zone (Wang et al., 2013). Hussain et al. (2019) reported that the droplet deposition density decreased with the increase in flight height and flying speed. The same trend was reported by Vitoria et al. (2023) and Wang et al. (2013). A similar study was reported on cotton: 15.91, 12.55, and 8.46 on the top, middle, and bottom canopy at a flight height and a flying speed of 1.5 to 2 m and 3 to 3.5 m/s, respectively (Yao et al., 2017). There was a higher *R* ^2^ of 0.9926 for the pigeon pea crop, which indicates that the 99.26% variability of the response could be explained, which is statistically accurate (Askari et al., 2021).

**Table 7:** Analysis of variance of droplet density at different flight heights and flying speeds.

Sources	DF	Sum of squares	Mean squares	*F*-calculated	*p*-value
Replication	2	0.796			
Flight height, m	2	33.88	161.14	55.06	0.0000*
Flying speed, m/s	2	90.50	43.25	15.95	0.0000*
Canopy position	2	67.79	33.89	11.58	0.0000*
Flight height × Flying speed	4	7.27	17.82	5963	0.0000*
Flight height × Canopy position	4	8.75	2.19	7.33	0.00009*
Flying speed × Canopy position	4	5.37	1.34	4.49	0.00341*
Flight height × Flying speed × Canopy position	8	2.074	0.259	0.86	0.5494NS
error	52	15.54	0.299		
Total	81	231.97			
CD@5% = 0.8956, CV = 4.49					

CV, coefficient of variation, *p < 0.05: significant at 5% level of significance; NS, non-significant.

Moreover, the smaller the CV, the better the uniformity of droplet distribution (Yao et al., 2017). The CV in the target zone (central line) was 4.49 while CD was 0.8956. There is a direct relationship between UAV flying speed and droplet density. From the study, it was observed that higher flying speeds produced lower droplet density, while slower flying speeds produced higher droplet density. This link could be explained by the smaller droplets’ propensity to form more quickly and their lower droplet density (Biglia et al., 2020).

The droplet density deposited per unit area was more than 15 droplets cm^−2^ and a spray coverage of more than 1% indicates better and effective application of agrochemicals through UAV (Ahmad et al., 2020). Moreover, other important parameters influence the UAV operation such as the heavy downwash produced by the UAV propeller, environmental parameters (temperature, relative humidity, and wind speed), pesticide characteristics (density and viscosity), and the size or shape of trees (Tang et al., 2018; Cidoncha et al., 2015; Vanitha et al., 2016; Zhang et al., 2017). Droplet densities of more than 20, 30 to 40, and 30 to 70 droplets cm^−2^ for insecticides, herbicides, and fungicides were considered effective spray application through UAVs (Ahmad et al., 2020; Biglia et al., 2020).

### 3.4 Droplet size

**Figure 10:**
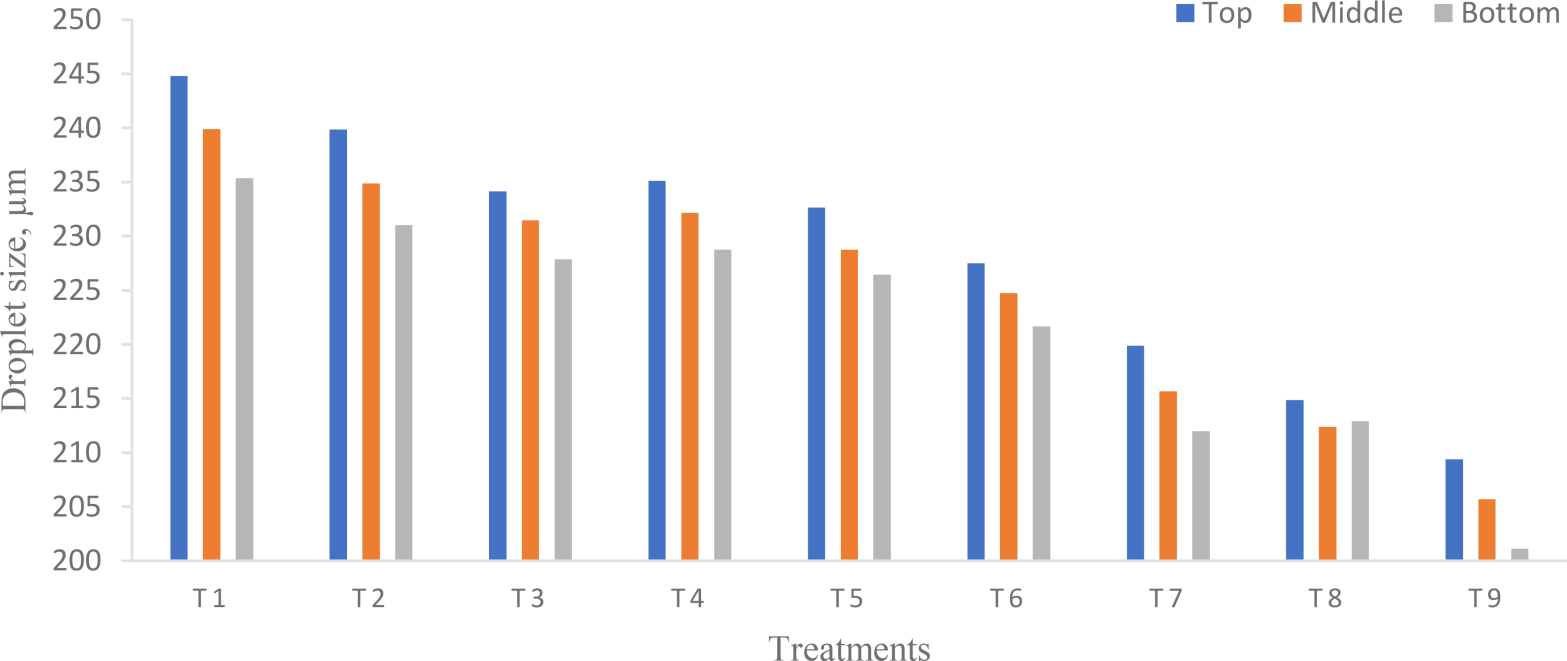
Effect of flight height and flying speed on droplet size at different canopy positions.

Among the most important factors that need to be evaluated when spraying to assess technology are droplet size and droplet distribution uniformity (Qin et al., 2016). The droplet size was influenced by the flight height and flying speed of UAVs (**Figure 10**).

ANOVA (**Table 8**) shows that there was a significant difference in droplet size on each canopy of pigeon pea crop at different flight heights and flying speeds (*p* < 0.05). From **Figure 10**, the highest droplet size was observed on the top canopy as compared to the middle and bottom canopy on the pigeon pea crop in the target zone. The highest and lowest droplet size deposited for treatments T_1_ and T_9_ were 244.80 and 209.38 µm on the top, 239.88 and 205.70 µm on the middle, and 235.37 and 201.13 µm on the bottom canopy of the pigeon pea crop, respectively. Similarly, Cao et al. (2021) reported that the droplet size was 25, 119, and 111 µm on the top, middle, and bottom canopy at a flight height and a flying speed of 1 m and 1.5 m/s, respectively. The uniformity of droplets was attributed to the relative span of the droplets. **Table 9** shows the relative span factor for various sampling sites at different treatments. The maximum and minimum relative span in the target zone (central line) for treatments T_1_ and T_9_ were 0.98 and 0.66 on the top, 0.96 and 0.63 on the middle, and 0.93 and 0.60 on the bottom canopy, respectively. The higher *R* ^2^ of 0.9960 for the pigeon pea crop canopy indicates that the 99.60% variability of the response could be explained, which is statistically accurate (Hussain et al., 2019). Moreover, the smaller the CV, the better the uniformity of droplet distribution (Tang et al., 2018). The CV in target zone (central line) was 1.65 while CD was 0.8454.

**Table 8:** Analysis of variance on the effect of flight height and flying speed on droplet size.

Sources	DF	Sum of squares	Mean squares	*F*-calculated	*p*-value
Replication	2	0.566			
Flight height, m	2	82.491	41.46	15.95	0.0000*
Flying speed, m/s	2	182.047	54.24	20.73	0.0000*
Canopy position	2	631.405	31.704	11.57	0.0000*
Flight height × Flying speed	4	53.962	13.491	51.60	0.0000*
Flight height × Canopy position	4	23.688	5.922	22.10	0.0000*
Flying speed × Canopy position	4	14.504	3.626	13.77	0.0000*
Flight height × Flying speed × Canopy position	8	40.276	5.035	19.130	0.0000*
error	52	13.685	0.263		
Total	81	1042.64			
CD@5%= 0.8454, CV = 1.65, *R* ^2^ = 0.9860					

CV, coefficient of variation, *p < 0.05: significant at 5% level of significance; NS, non-significant.

**Table 9:** Relative span factor at various sampling sites for different treatments.

Treat	Target zone			Non-target zone					
	Central line			2 m			4 m		
	Top	Middle	Bottom	Top	Middle	Bottom	Top	Middle	Bottom
T1	0.98	0.96	0.93	0.95	0.91	0.89	0.91	0.88	0.85
T2	0.94	0.92	0.89	0.91	0.88	0.84	0.85	0.81	0.77
T3	0.88	0.85	0.81	0.85	0.82	0.79	0.78	0.75	0.72
T4	0.83	0.79	0.76	0.81	0.79	0.76	0.72	0.69	0.65
T5	0.81	0.79	0.75	0.78	0.75	0.72	0.75	0.72	0.68
T6	0.75	0.71	0.68	0.72	0.69	0.65	0.69	0.66	0.62
T7	0.71	0.68	0.64	0.66	0.62	0.59	0.61	0.57	0.54
T8	0.69	0.66	0.63	0.65	0.62	0.59	0.61	0.58	0.55
T9	0.66	0.63	0.6	0.63	0.61	0.58	0.60	0.56	0.51

In the spraying operation, droplets are not required on a priority basis. However, droplet size is a more significant parameter during the spraying operation. A droplet size smaller than 50 µm would be easy to drift from the target zone, while a droplet size larger 300 µm had difficulty penetrating the bottom canopy of crops. A number of factors influence the spraying operation, and droplet size is one of them. The ideal droplet size for the spraying operation was 50 to 300 µm (Qin et al., 2016). For spraying insecticide and fungicides, the required droplet size was 150 to 300 µm (Qi et al., 2023). Conversely, larger size droplets considerably lower the drift as compared to finer droplets (Al Heidary et al., 2014). Also, the droplet size was found to be higher on the top canopy of plants as compared to the middle and bottom canopy in the target zone (central line) because increased flight height and flying speed generate strong downwash and increased weather wind speed leads to decreased droplet size and the heavy canopy of the pigeon pea crop creates problems for large droplets in terms of penetrating the bottom canopy with increased flight height and flying speed. Similar results were reported by Tian et al. (2020). **Figure 10** shows that the flight height and flying speed had a negative effect on droplet size. Similarly, Qi et al. (2023); Wang et al. (2023b), and Cao et al. (2021) reported the same result.

### 3.5 Spray drift characteristics of UAV at various sampling sites for different treatments

**Figure 11:**
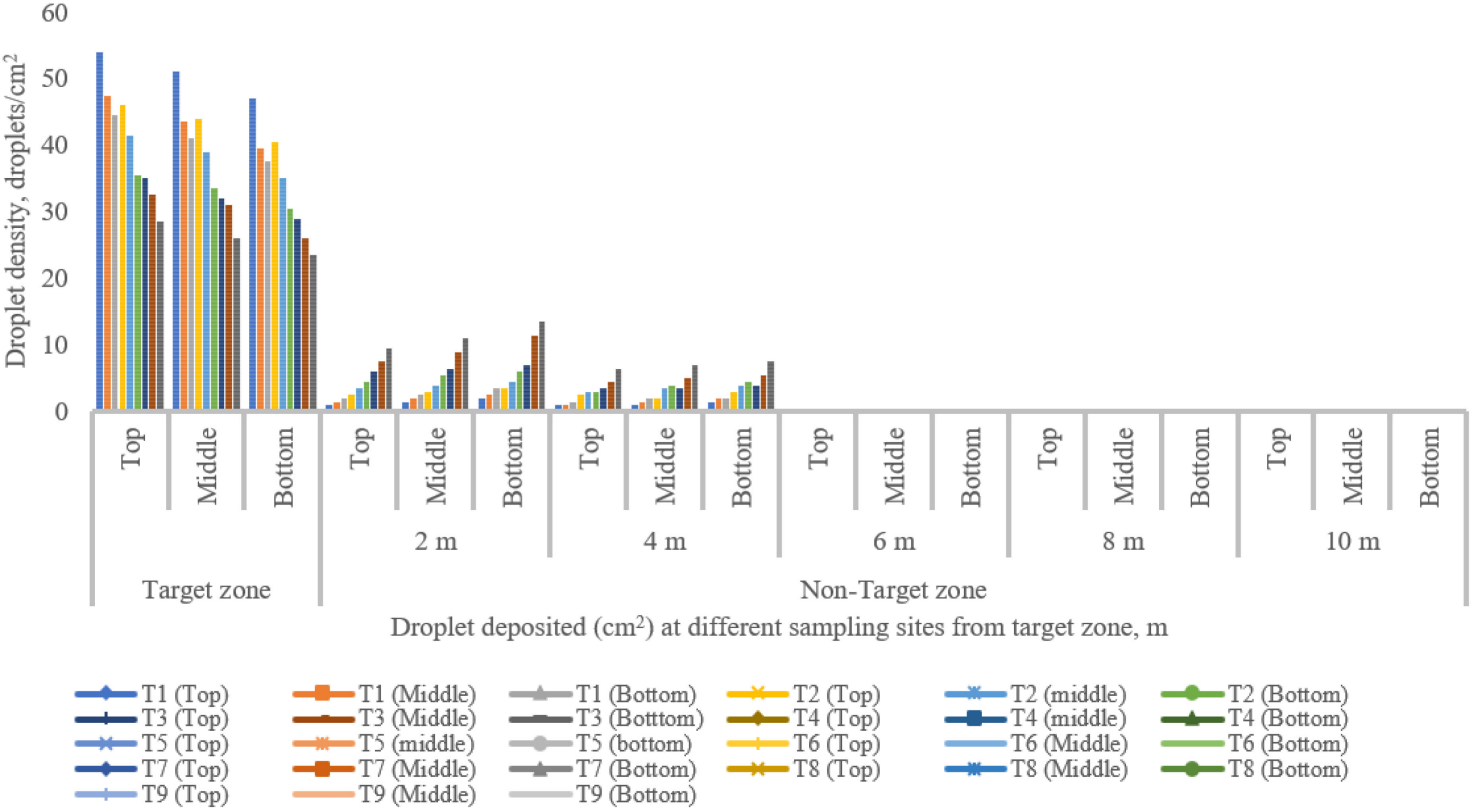
Droplets drifted from the target to the non-target zone at different collection points during various runs.

**Figure 12:**
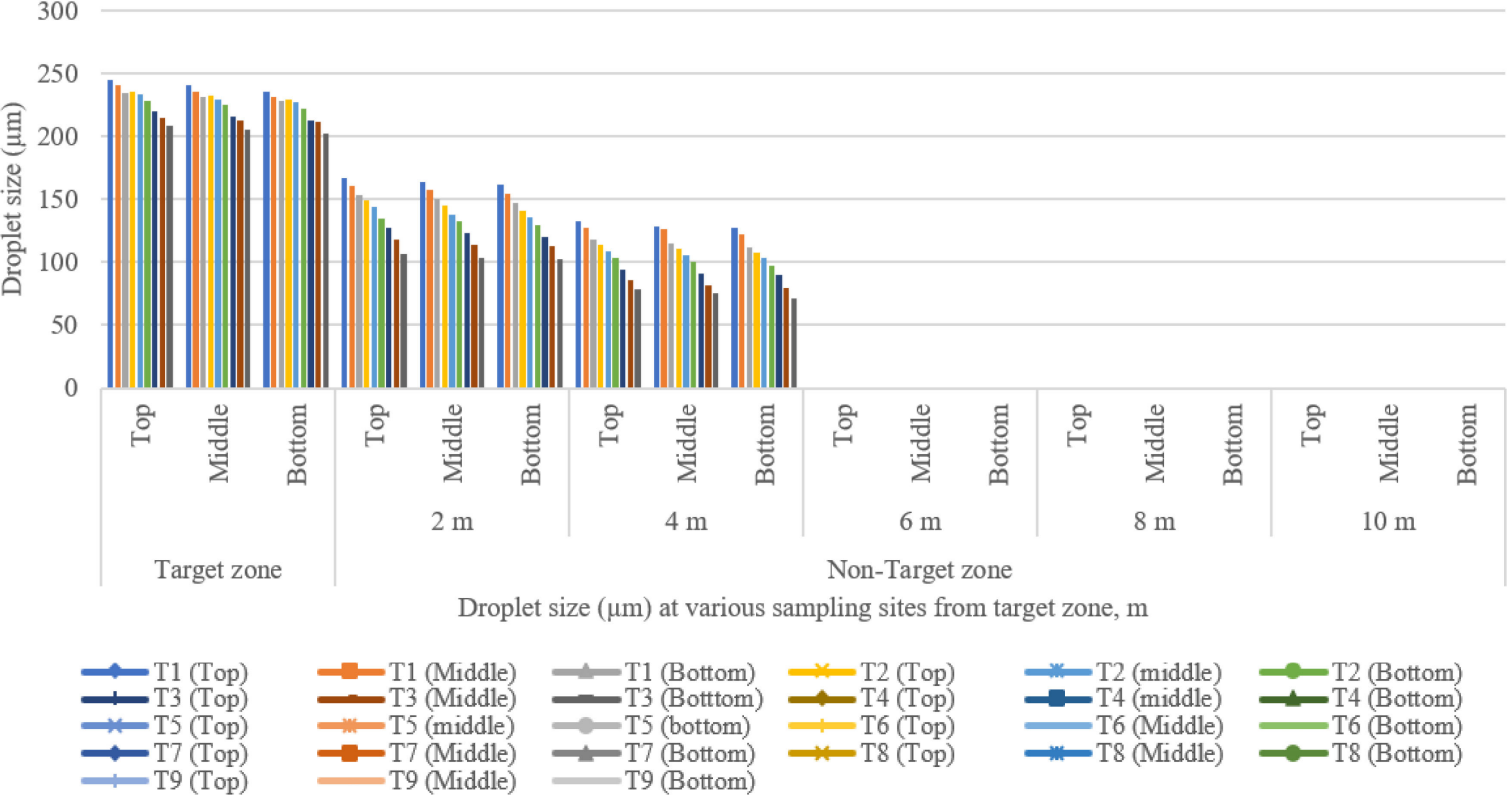
Droplet size from the target to the non-target zone at different collection points during various runs.

**Figure 13:**
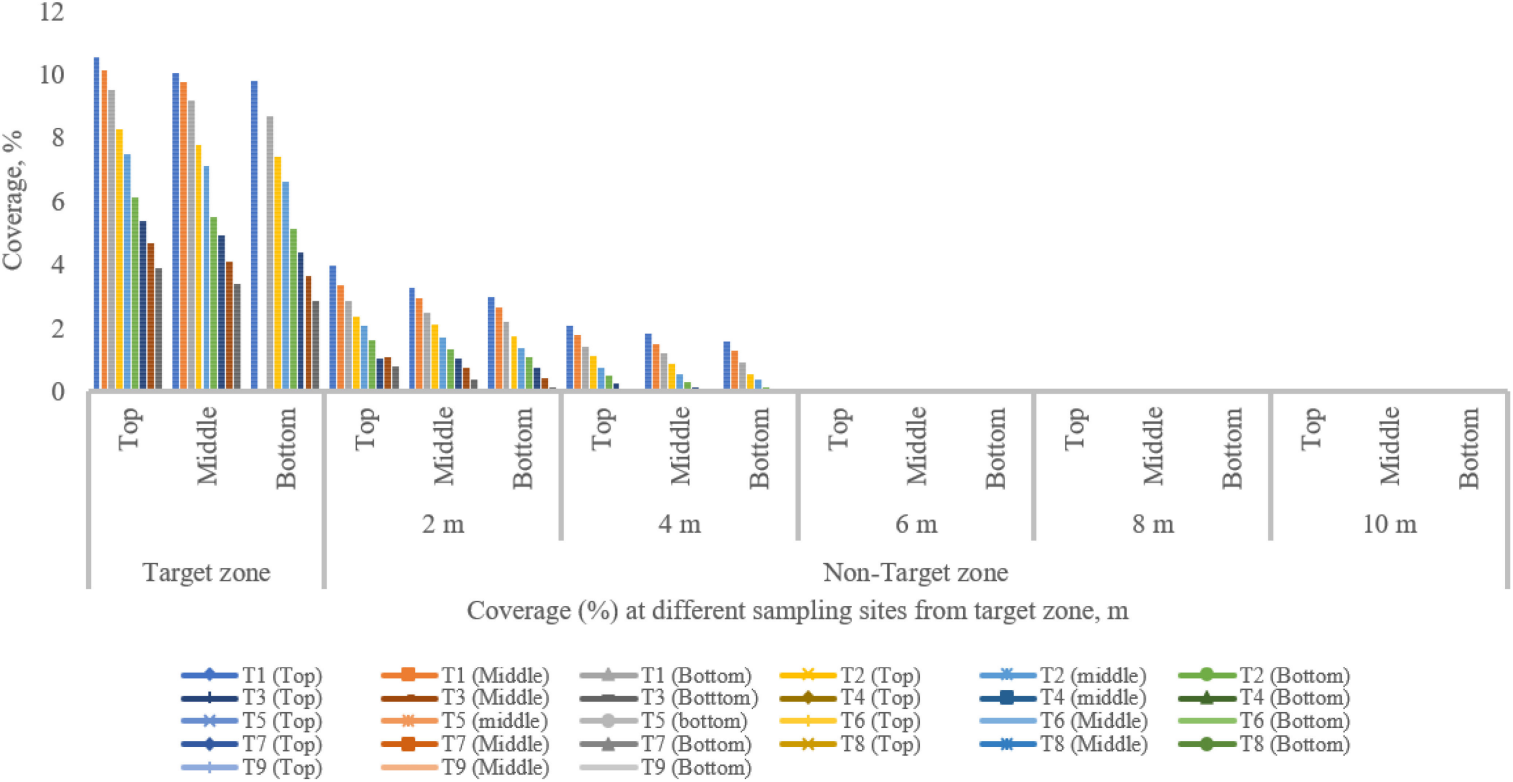
Coverage from the target to the non-target zone at different collection points during various runs.

**Figure 14:**
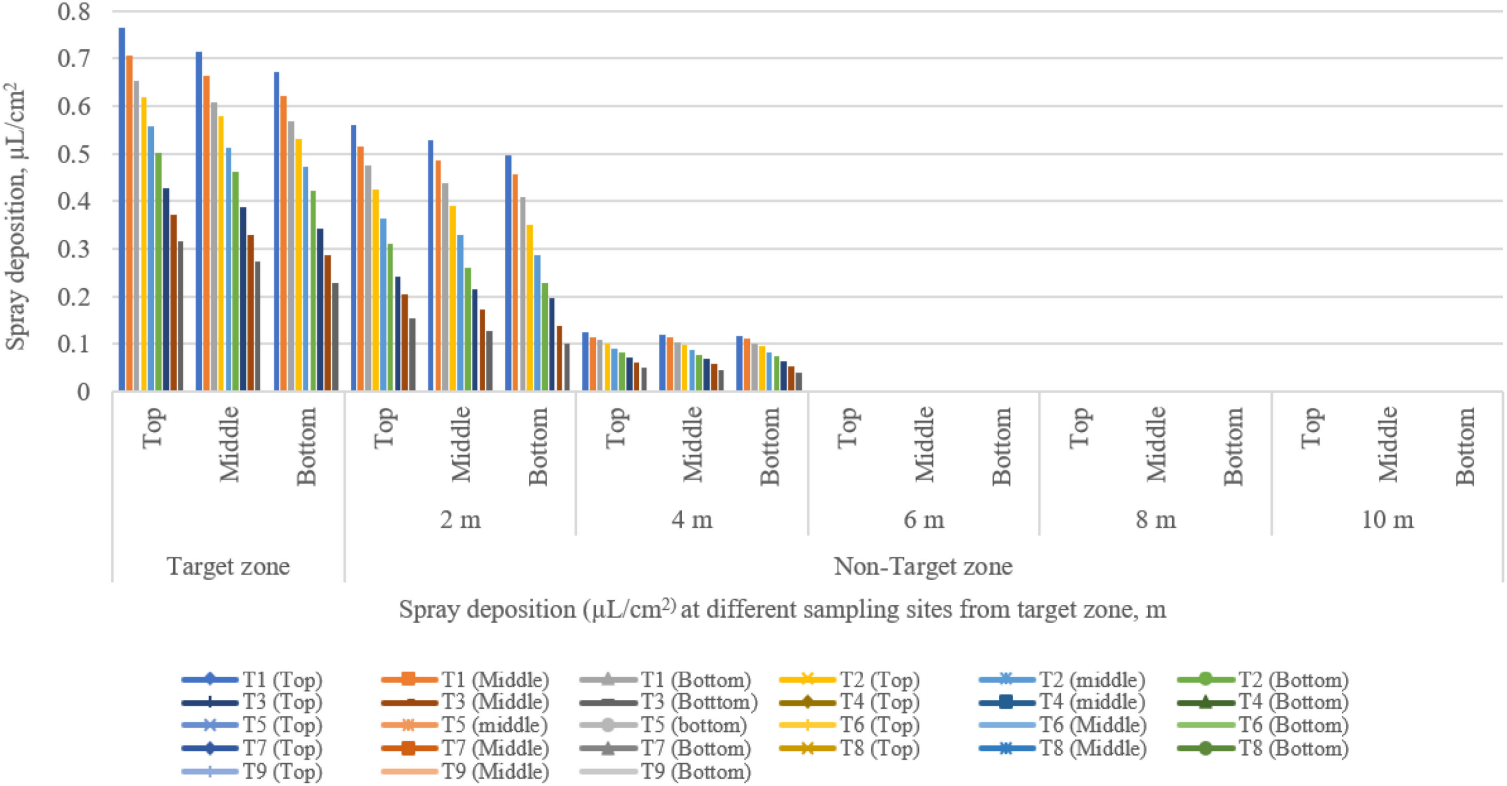
Spray deposition from the target to the non-target zone at different collection points during various runs.

The coverage and droplet distribution deposition in both target and off-target zones were assessed immediately after the spraying operation by using WSP, as illustrated in **Figure 8** (Zhu et al., 2011). **Figures 11**–**14** show the assessment of droplet density, droplet size, coverage, and spray deposition on each canopy in the target and off-target zones, respectively. The droplet deposition distribution, droplet size, coverage, and spray deposition were higher in the target zone (central line), but these parameters decreased as distance increased, as shown in **Figure 8**.

Droplet deposition and distribution are among the most significant characteristics to assess spray efficacy. **Figure 11** depicts the droplets deposited in a unit area in the target and off-target zones under different treatments. The highest and lowest droplet density deposited on the top, middle, and bottom canopy of pigeon pea crop in the center line (target zone) were 54.00, 50.17, and 46.33 droplets/cm^2^, and 29.33, 26.50, and 23.67 droplets/cm^2^ for treatments T_1_ and T_9_, respectively. The maximum number of droplets was deposited below the UAV (target zone) due to the downwash air produced by the UAV’s propeller (Al Heidary et al., 2014; Wen et al., 2019; Zheng et al., 2018). The lowest number of droplets deposited in an off-target zone in a unit area on the top, middle, and bottom canopy of the pigeon pea crop was 1.00, 1.50, and 2.00 droplets/cm² at 2 m; 1.00, 0.67, and 1.00 and 1.50 droplets/cm² at 4 m; and no droplets were found after 4 m distance from the target zone at treatment T_1_. The highest numbers of droplets deposited in off-target zones in a unit area on the top, middle, and bottom canopy of the pigeon pea crop were 9.50, 10.00, and 13.50 droplets/cm² at 2 m; 6.50, 7.00, and 7.50 droplets/cm² at 4 m; and no droplets were observed after 4 m distance from the target zone at treatment T_9_. The above assessment shows that operational and meteorological parameters such as wind speed and direction influence droplet deposition distribution. **Figure 11** clearly shows that increasing flight height and flying speed increases the distance droplets travel from the target to the non-target zone. Reducing the flying height of a UAV spraying will decrease the distance the droplets travel in the air before being deposited in the target zone (Yang et al., 2019).

The main factors influencing spray drift are wind speed, wind direction, flight height, flying speed, nozzle type, the arrangement of the nozzle, and downwash produced by the UAV’s propeller, which decreases the chance of droplet distribution and penetration on the target or crop leaves. Similar results were reported by Bueno et al. (2017). Smaller droplet size nozzles exhibit more drift challenges than larger droplet size nozzles (Wang et al., 2023a). Spraying operations have a substantial influence on droplet deposition distribution properties such as droplet size and spray deposition. To provide a successful effect, a spray droplet must exceed a certain threshold (Zhu et al., 2011). The largest and smallest sizes of droplet observed on the top, middle, and bottom pigeon pea canopy in the central line were 244.80, 239.88, and 235.37 µm and 209.38, 205.70, and 201.13 µm for treatments T_1_ and T_9_, respectively. The largest size of droplets observed on the top, middle, and bottom canopies of the pigeon pea crop in the off-target zone were 166.25, 163.03, and 161.00 µm at 2 m; 132.65, 128.47, and 126.92 µm at 4 m; and no droplets were found after 4 m distance from the target zone at treatment T_1_. The smallest sizes of droplets observed on the top, middle, and bottom canopy of the pigeon pea crop in the off-target zone were 106.55, 103.32, and 101.79 µm at 2 m; 78.25, 75.39, and 71.06 µm at 2 m; and no droplets were found after 4 m distance from the target zone at treatment T_9_. **Table 9** depicts the relative span factor that determines the uniformity of droplet size distribution. The highest and lowest relative spans in the target zone were 0.98, 0.96, and 0.93, and 0.66, 0.63, and 0.60 for treatments T_1_ and T_9_. Because of crosswind fluctuations, the total number of droplets deposited on the right side off the target zone was greater than that on the left side off the target zone. Furthermore, droplet drift and deposition occurred more to the right of the target zone. This is mostly determined by the external wind speed blowing from the left to the right side of the flow path. **Figure 12** illustrates that as flight height and speed increase, droplet size reduces and scatters across the target zone (right side of the central line). **Figure 12** shows that larger droplets (>50 µm) are deposited directly below the UAV or in the target zone, while smaller droplets (<50 µm) travel long distances and are deposited after losing their kinetic energy.

**Figure 13** shows spray coverage in both the target and non-target zones for various treatments. The maximum and minimum coverage of the top, middle, and bottom canopy of the pigeon pea crop in the central line (target zone) were 10.52%, 10.05%, and 9.78%, and 3.88%, 3.39%, and 2.86% for treatments T_1_ and T_9_, respectively. A significant variation was noticed between the center line and the observation line. Furthermore, there is a synergistic relationship between coverage and spray droplet deposition. The droplet deposition uniformity achieved by adjusting UAV operation parameters is critical for decreasing spray drift and enhancing penetrability.

**Figure 8** depicts the WSP card threshold pictures that were obtained immediately after the spraying operation for all treatments, including in the target and off-target zones, and shows that spray coverage dropped as the distance from the target zone to the off-target zone increased because of variation in wind speed, direction of wind speed, and operational parameters of UAV. After the spraying operation, the WSPs were collected manually immediately in both the target and off-target zones (**Figure 8**). Spray coverage reduced as flight height and flying speed increased. Similar results were reported by Ahmad et al. (2020). The maximum coverage on the top, middle, and bottom canopy of the pigeon pea crop in the off-target zone was 3.95%, 3.25%, and 2.98% at 2 m and 2.06%, 1.92%, and 1.57% at 4 m, respectively, with no coverage detected after 4 m distance from the target zone after treatment T_1_. The lowest coverage on the top, middle, and bottom canopy of pigeon pea crop in the off-target zone was 0.78%, 0.38%, and 0.12% at 2 m from the target zone; 0.022%, 0.0045%, and 0.0013% at 4 m from the target zone; and no coverage was recorded beyond 4 m at treatment T_9_. From the above discussion, as flight height and speed rise, coverage in both the target and off-target zones diminishes. Similarly, Lou et al. (2018) investigated the use of UAVs to manage cotton aphids and spider mites and discovered that spray deposition and coverage are affected by flight height and speed.

Spray deposition in the target and non-target zone for various treatment is presented in **Figure 14**. The highest and lowest spray deposition on the top, middle, and bottom canopy of the pigeon plant in the central line (target zone) were 0.765, 0.714, and 0.672 µl/cm^2^, and 0.315, 0.272, and 0.215 µl/cm^2^ for treatments T_1_ and T_9_, respectively. There was a significant difference observed in the central line along the observation line. The highest spray deposition on the top, middle, and bottom canopy of pigeon plants in the off-target zone was 0.559, 0.527, and 0.495 µl/cm^2^ at 2 m; 0.125, 0.119, and 0.116 µl/cm^2^ at 4 m distance from target zone; and no spray deposition was found after 4 m distance from the target zone at treatment T_1_. The lowest spray deposition on the top, middle, and bottom canopy of pigeon plants in the off-target zone was 0.154, 0.126, and 0.101 µl/cm^2^ at 2 m; 0.0049, 0.0045, and 0.0041 µl/cm^2^ at 4 m distance from target zone; and no spray deposition was found after 4 m distance from target zone at treatment T_9_. The above discussion shows that with the increase in flight height and flying speed, spray deposition decreases in both the target zone and the non-target zone.

### 3.6 Field performance parameters

Various field performance parameters were measured during each spraying operation. The discharge rate of each nozzle was 0.850 L/min and the total discharge rate of four nozzles was 3.4 L/min. The highest theoretical field capacity, effective field capacity, field efficiency, and application rate were 4.32 ha/h, 2.62 ha/h, 60.64%, and 77.86 L/ha observed at a flight height and a flying speed of 1.5 m and 2 m/s, respectively (**Table 10**). Various time losses such as filling tank, replacing battery, adjustment of propeller and nozzle, turning, takeoff, and landing were considered during each spraying operation of UAV. The noise (measured by HTC SL1350 manufactured by MEXTECH, India) observed during the spraying experiment ranges from 59.75 to 65.75 dB(A). For safe, prolonged, and comfortable operation, noise should not be more than 85 dB(A) for 8 h per day (Sharma and Mukesh, 2021). The NABARD guideline was followed for the calculation of the economic cost of UAV (Anonymous, 2023). The cost of operation and payback period of UAV were 649.17 Rs/ha and 1.21 years, respectively.

**Table 10:** Various field parameters measured during experiments.

Flight height, m	Flying speed, m/s	UAV			Noise dB(A)	Application rate of UAV, L/ha
		TFC, ha/h	EFC, ha/h	FE, %		
1.5	2	4.32	2.62	60.64	62.02	77.86
	2.5	5.76	3.55	61.63	63.45	57.46
	3	7.2	4.62	64.67	65.75	44.15
2	2	4.32	2.54	58.79	61.77	80.31
	2.5	5.76	3.32	57.64	62.72	61.44
	3	7.2	4.28	59.44	63.11	47.66
2.5	2	4.32	2.42	56.02	59.75	84.29
	2.5	5.76	3.18	55.20	60.57	64.15
	3	7.2	3.85	53.47	61.39	52.98

### 3.7 Control efficacy of thrips

**Figure 15:**
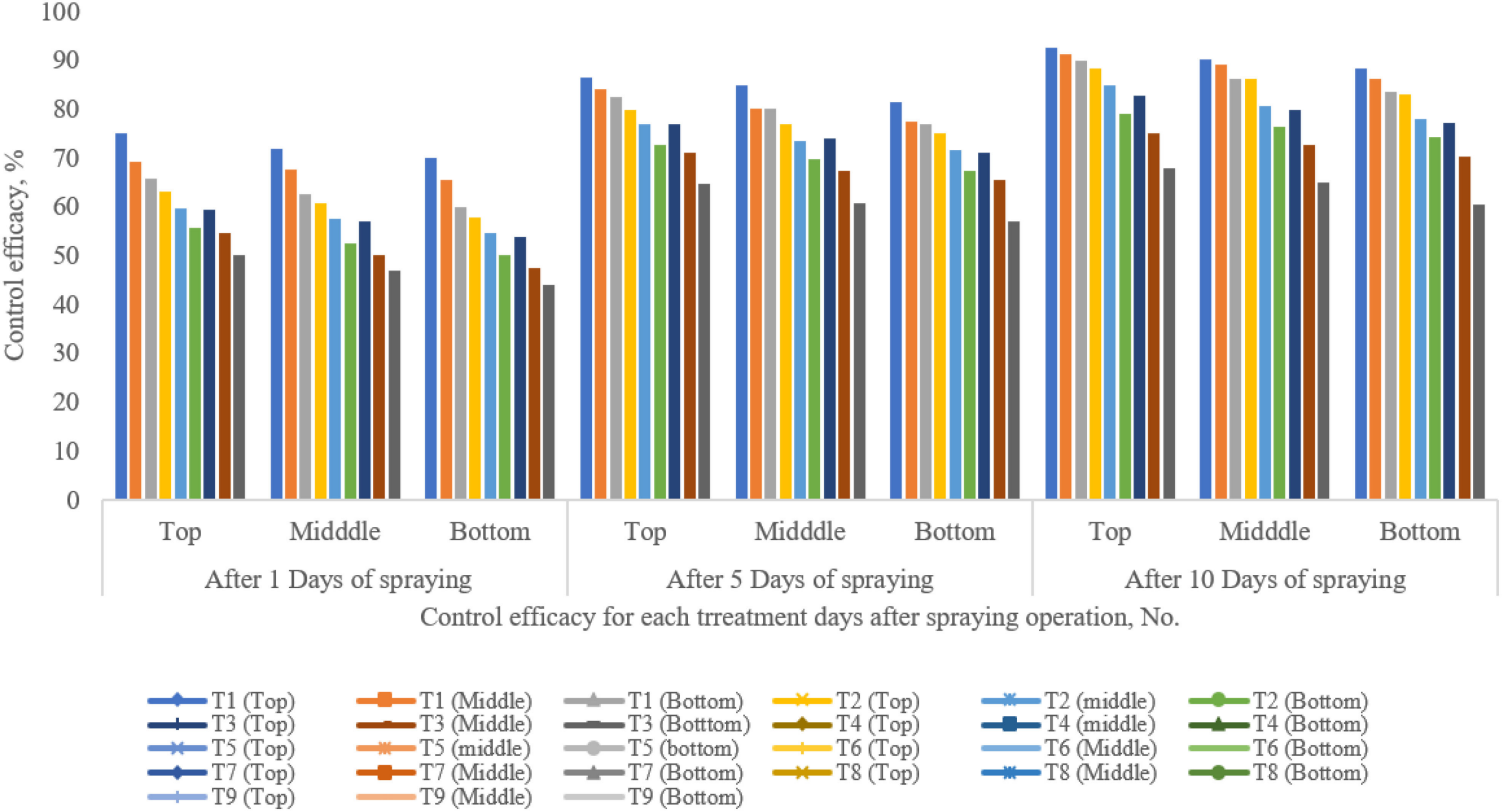
Thrips control efficacy in pigeon pea crops.

The efficacy of UAV sprayers for controlling pigeon pea pests in terms of thrips reduction was determined. The control efficacy of thrips on the top, middle, and bottom pigeon pea canopy plant was 74.95%, 71.85%, and 69.65%; 86.46%, 84.76%, and 81.42%; and 92.45%, 90.12%, and 88.11% after 1, 5, and 10 days of spraying experiment, respectively. From the above results, it was observed that the control efficacy of thrips was the highest after 10 days of spraying experiment at a flight height and a flying speed of 1.5 m and 2 m/s, respectively, as compared to other treatments (**Figure 15**). Therefore, the control efficacy of spider thrips was decreased with the increase of flight height and flying speed. The droplet density deposited on the target zone is one of the most crucial factors for determining insecticide efficiency. At a flight height of 1.5 m and a flying speed of 2 m/s, the control efficacy could be the highest due to more droplet deposition as compared to other treatments. Similar results were reported by Qin et al. (2016) and Lou et al. (2018).

## 4 Conclusions

The operational parameters of a UAV-based spraying system were optimized in the pigeon pea crop. The following are the conclusions drawn from the study:

1. The best spray performance was observed at a flight height of 1.5 m and a flying speed of 2 m/s.
2. The maximum spray performance parameters on the top, middle, and bottom of the pigeon pea canopy, i.e., spray coverage (10.53%, 10.09%, and 9.78%), spray deposition (0.764, 0.714, and 0.672 µl/cm^2^), droplet density (54.00, 50.17, and 46.33 droplets/cm^2^), and droplet size (244.80, 239.88, and 235.37 µm), respectively, were obtained at a flight height of 1.5 m (above crop canopy) and a flying speed of 2 m/s.
3. The droplets drifted from the target zone up to 4 m in the non-target zone, but negligible spray deposition was observed after 2 m from the target zone at a flight height of 1.5 m (above crop canopy) and a flying speed of 2 m/s.
4. The field capacity, field efficiency, and application rate of UAV were 2.62 ha/h, 60.64%, and 77.86 L/ha, respectively.
5. The maximum control efficacy of thrips on the top, middle, and bottom pigeon pea canopy was 92.45%, 90.12%, and 88.11%, respectively, after 10 days of spraying experiment.
6. The present study provides recommendations to manufacturers, farmers, and UAV operators for more effective and efficient spraying on pigeon pea crop by using the quadcopter UAV at a flight height of 1.5 m above the crop canopy and a flying speed of 2 m/s.

## Data Availability

The original contributions presented in the study are included in the article/supplementary material. Further inquiries can be directed to the corresponding author.
